# Acute parenchymal hemorrhage of three cases report after burr hole drainage of chronic subdural hematoma

**DOI:** 10.11604/pamj.2018.31.140.13982

**Published:** 2018-10-25

**Authors:** Yang Wang, Xiangping Wei

**Affiliations:** 1 Department of Neurosurgery, Anhui Provincial Hospital Affiliated to Anhui Medical University, 17 Lujiang Road, Hefei 230001, China

**Keywords:** Acute parenchymal hemorrhage, chronic subdural hematoma, burr hole drainage

## Abstract

Chronic subdural hematoma (CSDH) is one of the most common neurological diseases, which mainly occurs among elderly people and usually develop after minor head injuries. Over the years, a simple burr hole evacuation of the hematoma has been accepted as the widespread method for most cases of CSDH, but acute parenchymal hemorrhage is a rare and deadly complication after surgery. We report three elderly cases of post-operative parenchymal hemorrhage and analyse the underlying factors and formulate relevant strategies in this article. Three advanced age patients had been admitted to our department with gradually increasing headache and limb activity disorder urgently and underwent an emergency operation of burr hole drainage of CSDH in frontal-temporal region after preoperative evaluations and examinations. Unfortunately, acute post-operative parenchymal hemorrhage occurred in three advanced age patients. Ultimately, the patients achieved satisfying outcome with no significant neurological deficit through conservative treatment. The exact mechanism of such uncommon complications are difficult to explain and remain poorly understood. Advanced age, hypertension, amyloidosis, high perfusion triggered by rapid hematoma release, cerebrospinal fluid (CSF) loss, oral anticoagulant, primary disease aggravation were the main mechanisms which were speculated in our report. Simultaneously, positive measures could be adopt to prevent this rare complication.

## Introduction

Chronic subdural haematoma (CSDH) is one of the most common neurosurgical conditions and is especially prevalent among elderly individuals [1]. Behavioural disturbance and neurological deficits are the most common first symptoms. Head trauma, advanced age, coagulopathies, and therapeutic anticoagulation are considered as risk factors [2]. In addition, intracranial hypotension due to cerebrospinal fluid (CSF) loss and the age-related increase of subdural space and subarachnoid cisterns may also accelerate CSDH formation [3]. Current management strategies of CSDH remain widely controversial. These strategies include surgical techniques such as single burr-hole drainage under local anesthesia, double burr-hole evacuation technique, formal craniotomy. Burr-hole craniectomy combined with subdural drainage is a relatively safe and effective management option [4, 5]. Neuroendo-scopic techniques have also been proposed for multiloculated or septated haematomas, but are rarely recommended routinely [6]. In our institution, bur hole craniostomy under local anesthesia has been the treatment of choice for patients with CSDH. Postoperative CSDH complications include recurrence, acute subdural hematoma, acute intracranial bleeding, chronic subdural collection of fluid, epilepsy, subdural empyema and tension pneumocephalus [7-9]. Previous reports have indicated acute parenchymal hemorrhage may occur in routine craniotomy and is related to a poor clinical outcome [10]. In our report, acute parenchymal hemorrhage all occurred in 3 cases after burr hole drainage. In our research, 135 CSDH patients?33 cases were over 70 years old) who admitted to Department of Neurosurgery, Anhui Provincial Hospital Affiliated to Anhui Medical University from August 2015 to October 2016 were retrospectively analysed. Unfortunately, acute post-operative and parenchymal hemorrhage occurred in advanced age patients, with an incidence of 9.9%. This study was done to investigate the causes of this uncommon complications and formulate relevant therapeutic strategies.

## Patient and observation

**Figure 1:**
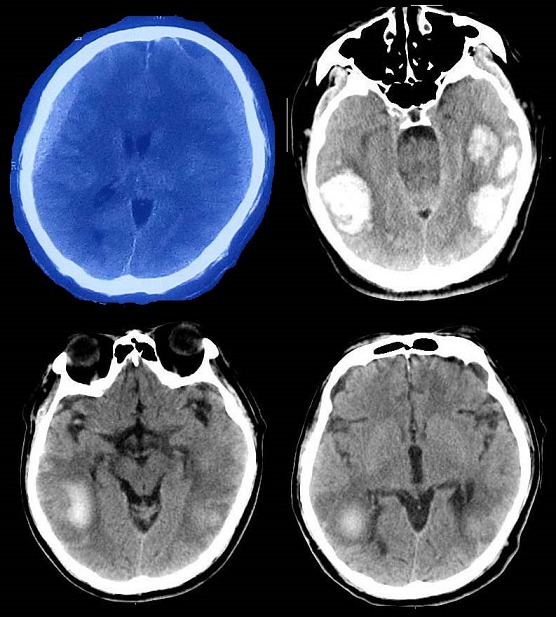
Preoperative CT showed bilateral CSDH (chronic subdural hematoma); postoperative CT show multiple parenchymal hemorrhage; follow-up CT show hematoma absorption

**Case 1:** An 71-year-old male patient had been admitted to our department with two-week history of gradually increasing headache and slight limb activity disorder and a 2-month history of minor head trauma as a result of fall to the ground. Cranial CT revealed bilateral CSDH. There was hypertension and multiple lacunar infarction were the systemic diseases which were under normal control (Table 1). Chest radiography, electrocardiogram and the blood system examinations were completed preoperatively. Fortunately, the major data was not exceptional remarkably and preoperative coagulation parameters were within normal limits. The patient underwent an urgent operation of burr hole drainage of CSDH in bilateral frontal-temporal region. During the operation, subdural hematoma launched from bone hole after the opening of the dura in spite of over high intracranial pressure (ICP). The procedure was completed without any obvious accident and the vital signs remained stable during operation. Eventually, a subdural closed system of drainage was placed when intracranial pneumatosis was eliminated absolutely. Because of the exacerbation of conscious disturbance, emergent CT scan was taken at 2 hours postoperatively and revealed multiple parenchymal hemorrhage. Postoperative recovery was uneventful. Surgical intervention was not adopted, and the patient kept to conservative treatment including continuous neurological observation for 6 days in the NICU. The hospitalization time was prolonged to 20 days because of mental disturbance after the operation which was corrected successfully in that interval. Finally, the patient was discharged from hospital with no neurological deficit except for slight language barriers. At 2-month follow-up no abnormality was noted, and the CT scan revealed hematoma was absorbed completely (Figure 1).

**Table 1:** pre-operative and post-operative data of 3 cases

Items	Case 1	Case 2	Case 3
Age	75y	81y	83y
Gender	Male	Male	Male
Side	Bilateral	Right	Right
Presention	Headache, limb, activity disorder	Limbs weakness	Dizziness and Headache
Primary disease	Hypertension, lacunar infarction	Cerebral infarction, coronary heart disease	Hypertension, COPD, asthma, ischemic heart disease
Anticoagulant	None	Rivaroxaban	None
Operation method	Burr hole drainage	Burr hole drainage	Burr hole drainage
Drainage amount (ml)	700	500	750
Extubation time (day)	4	4	5
Hospitalization time (day)	20	12	30

CSDH: chronic subdural hematoma; COPD: chronic obstructive pulmonary disease

**Figure 2:**
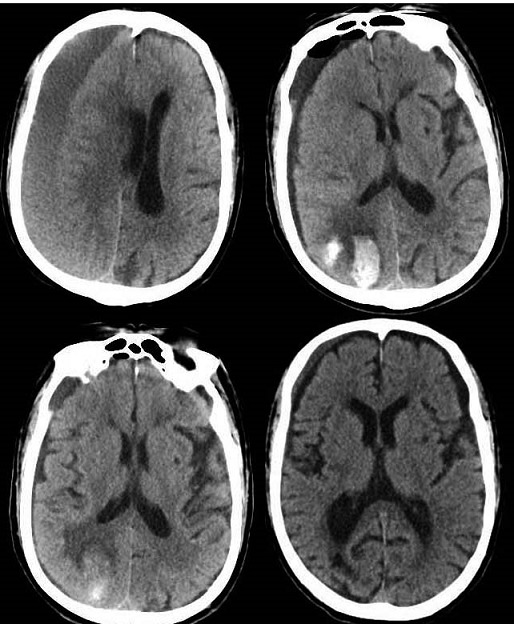
Preoperative CT showed a right equidensity CSDH (chronic subdural hematoma) and brain tissue compression; postoperative CT right occipital lobe hemorrhage; follow-up CT show hematoma absorption

**Case 2:** in case 2 we reported an 82-years-old man had been admitted to our department due to limbs weakness presented progressive aggravation with undergoing frequent falls in the previous month and a history of cerebral infarction, arterial hypertension and coronary heart disease with long-term oral rivaroxaban. The CT scan showed a right equi-density subdural hematoma and brain tissue compression. The neurologic evaluation revealed slow response and weakness of the left limbs (Table 1). The urgent evacuation of the subdural hematoma was performed with a single parietal burr-hole craniectomy on the right side without discontinuing oral rivaroxaban. A subdural drainage catheter was positioned in prefrontal region routinely. Surprisingly, we observed right visual field defect the following day and vital signs was remained within normal range all the time. The post-operative CT scan showed a bit of prefrontal pneumocephalus and right occipital lobe hemorrhage and no acute bleedings in ipsilateral subdural space. The drainage tube removal was delayed to the fourth day. Meanwhile, the CT findings occipital hematoma appeared absorbed gradually and did not require surgical intervention. The neurologic evaluation revealed a slight postural instability and for this reason that the patient was arranged to physical therapy. At two-month follow-up, the patient was able to walk unaided, and the review CT scan was normal (Figure 2).

**Figure 3:**
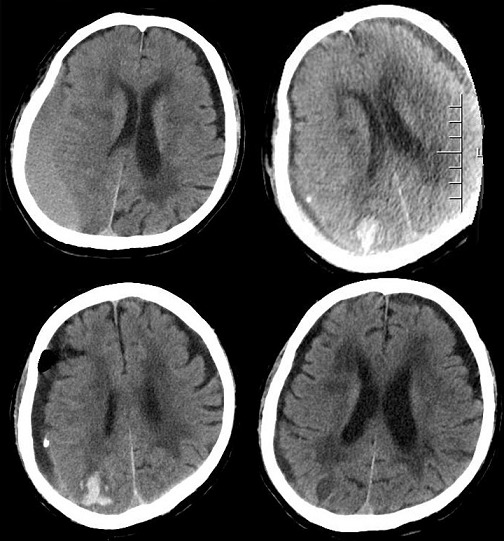
Preoperative CT showed a sizeable right CSDH (chronic subdural hematoma) which caused midline left shift; postoperative CT a small area occipital intraparenchymal hemorrhage; follow-up CT show hematoma absorption

**Case 3:** in case 3 we reported an 80-years-old man had been admitted to our department due to moderate dizziness and headache for 20 days with a history of hypertension, ischemic heart disease, chronic obstructive pulmonary disease (COPD) and asthma. Neurological assessment confirmed mental confusion and gait imbalance without focal deficits. The CT scan revealed a sizeable right chronic subdural hematoma which caused midline left shift (Table 1). The patient underwent an instant single burr-hole craniectomy and the right subdural hematoma evacuation was satisfactory largely. A subdural drainage apparatus was positioned routinely. On the second day after surgery, sudden airways spasm occurred and blood oxygen saturation presented unstable within a short time. Emergency tracheal incubation was implemented to maintain vital signs. Simultaneously, the drainage device was closed and a emergent CT scan was performed and revealed the presence of a small area occipital intraparenchymal hemorrhage. On the third day, the patient was transferred to the ICU and assisted respiration with ventilator postoperatively. Subsequently, the patient was required tracheotomy due to difficulty of removing trachea cannula within a short time. The drainage tube removal was delayed to the fifth day postoperatively. After one week, the patient successfully detached from the respirator and returned to general ward to continue anti-infective treatment for 10 days. Finally, the patient discharged from hospital with mild pulmonary inflammation, but therapeutic process was uneventful, and the total hospitalization time was up to one month. The two-month follow-up showed the patient was in good conditions and life-independent. The CT scan of the head revealed no recurrence (Figure 3).

## Discussion

Postoperative parenchymal hemorrhage generally occurs in operative region after tumor resection or hematoma clearance, mostly on account of inexact hemostasis. Subdural hematoma after drilling drainage operation of CSDH is the most common type of postoperative bleeding [11]. However, acute parenchymal hemorrhage occurrence after supratentorial burr hole drainage of CSDH is a kind of uncommon complications, which are difficult to explain and remain poorly understood at present [12, 13]. Moreover, what is amazing is that 3 cases we reported are all advanced age patients. Advanced age, hypertension, amyloidosis, high perfusion triggered by rapid hematoma release, CSF loss, oral anticoagulant, primary disease aggravation are the conceivable mechanisms currently debated. There was no doubt that the rate of multiple intracranial hemorrhage being similar to this condition in case 1 after burr hole evacuation of CSDH was percussive. The total hematoma volume was summed up to 40ml approximately without apparent midline shift and hematoma clearance combined with decompressive craniotomy should not be adopted under these circumstances due to mild disturbance of consciousness and neurologic defect. Although the actual pathophysiological mechanism still remained unclear, several possible causative factors have been considered, such as severe hypertension, amyloid degeneration, rapid brain decompression, shift of the intracranial contents during surgery and loss of CSF though analysing the whole process of diagnosis and treatment. Consequently, surgical procedure and occasion caused our attention. Blood pressure control, slow and concurrent evacuation of bilateral subdural hematoma was beneficial to prevent high perfusion and shift of the intracranial contents triggered by rapid hematoma release for bilateral CSDH patients. Once parenchymal hemorrhage occurred postoperatively, subdural drainage tube was raised or shut timely to stop hematoma expanding further. In addition, moderate dehydrator and coagulants application rather than hematoma evacuation was possible optimal choice for multifocal cerebral hemorrhage [14]. It goes without saying that therapeutic process was destined to be uneventful on condition that postoperative complications appeared, such as pulmonary infection and epileptic seizure. Fortunately, the therapeutic effect of this patient was satisfactory and discharged from hospital with no neurological deficit except for slight language barriers. It was well known that blood coagulopathy was suggested as a causative factor of postoperative intracranial bleeding all the time [15]. However, although there was no evident coagulation abnormality in pre-operative examination in case 2, we could not eliminate the underlying impact of oral anticoagulant drug, especially some new anticoagulants which are not acquainted so far absolutely.

In our case 2, there was no coagulation abnormality but a history of recent use of new anticoagulant [16]. Rivaroxaban, a new kind of direct inhibitors of factor Xa equipped with these advantages including fast acting time, short half-life and the low incidence of bleeding [17]. Most importantly, coagulation function monitoring was omitted during the period of oral drugs. Unfortunately, relevant deficiency was investigated in few literatures and rivaroxaban administration was not discontinued before surgery due to preoperative coagulation parameters being within normal limits. The assumption of coagulopathy caused by new anticoagulants should be considered as a risk factor resulting in ipsilateral occipital lobe hemorrhage. Therefore, when we encountered the CSDH patients with oral administration new anticoagulants, the specific procedures should be formulated. To begin with, the type and last medication time were realized, the necessity of emergency surgery was filtered strictly, and the mean time of drug discontinuance should be extended as long as possible for elective surgery [18]. Afterwards, laboratory examination, including APTT, PT, dTT and coagulation factors activities analysis were completed as quickly as possible and evaluated comprehensively [19]. Finally, blood products preparation, tranexamic acid, vitamin K1 application and supplement of PPSB were recommended regardless of existence of coagulation parameters abnormality [20]. To avoid infarction formation, new oral anticoagulants should be resumed as soon as possible after intracranial condition was stable.

In case 3, acute parenchymal hemorrhage took place followed by serious airways spasm exceeding our expectation. However, surgery acquired a success in this case sometimes, but the postoperative complications or primary diseases deterioration often make poor prognosis and the perioperative management of advanced age patients with CSDH brings us a challenge. Therefore, positive therapy of primary diseases, the management of respiratory tract, prophylactic drug use, early postoperative function exercise and multidisciplinary consultation can be prevented acute parenchymal bleeding induced by unexpectedly condition occurrence for advanced age patients. Despite all these analytical mechanisms are discussed through the above cases, no exact mechanisms have been confirmed to explain its occurrence properly. Although we cannot prevent bleeding events occurrence certainly after CSDH operation, we can decrease the incidence rate of acute parenchymal hemorrhage for hyper-aged CSDH patients which can't stand secondary brain injury provided that we conformed to normalized procedures.

## Conclusion

We reported three cases of uncommon intraparenchymal hemorrhage after burr hole craniotomy for CSDH. They are probably associated with Advanced age, hypertension, amyloidosis, high perfusion triggered by rapid hematoma release, cerebrospinal fluid (CSF) loss, oral anticoagulant, primary disease aggravation. Although CSDH drainage is considered as a minimally invasive surgical approach, neurosurgeons must be aware of this rare and deadly event. Correction of coagulation function, control of blood pressure and treatment of primary diseases are essential in order to prevent such complications before operation. Careful and gentle evacuation of the subdural hematoma without excessive head rotation is suggested to avoid rapid shift of intracranial contents and local hyper transfusion during operation. Continuous electrocardiograph monitoring, repeated evaluation of neurologic conditions, prompt radiological assessment, positive therapy of primary disease, volume of drainage control strictly and the height of drainage tube adjustment are mandatory after operation. Above all, when the uncommon complication occurs, conservative treatment is recommended intensively unless enormous intracranial hematoma make the patients unconsciousness, even brain hernia. Unfortunately, evacuation of hematoma and decompressing craniotomy were only adopted to save lives accompanied by poor prognosis.

## Competing interests

The authors declare no competing interests.
